# Curcumin Loaded Microsponges for Colon Targeting in Inflammatory Bowel Disease: Fabrication, Optimization, and *In Vitro* and Pharmacodynamic Evaluation

**DOI:** 10.1155/2014/340701

**Published:** 2014-07-01

**Authors:** Rashmi Sareen, Kavita Nath, Nitin Jain, K. L. Dhar

**Affiliations:** School of Pharmaceutical Sciences, Shoolini University, Bajhol, Solan, Himachal Pradesh 173229, India

## Abstract

The present study was aimed to develop and optimize the microsponges of curcumin for colon specific drug delivery in a view to bypass the upper gastrointestinal tract (GIT) for enhanced therapeutic effect. Microsponges were developed by quasi emulsion solvent diffusion method using 3^2^ full factorial design. Prepared microsponges were optimized in order to analyze the effects of independent variables (volume of ethanol and Eudragit L100) on the encapsulation efficiency, particle size, and drug release. The optimized formulation was subjected to *in vivo* study using acetic acid induced colitis model in rats. The F7 was selected as optimized formulation based on particle size of 41.63 *μ*m, % entrapment efficiency of 78.13%, and % cumulative drug release of 84.12%, and desirability factor of 0.83. Release studies revealed that microsponges prevented the premature release of curcumin in upper GIT and specifically released the drug at colonic pH. The drug release profile of F7 formulation was subjected to different kinetic models and based upon the best correlation coefficient (*r* ^2^ = 0.9927) the release was found to follow Higuchi model, which suggested diffusion as the main mechanism of drug release. Pharmacodynamic study showed that curcumin loaded microsponges causes a significant decrease in edema, necrosis, and hemorrhage of colon as compared to free curcumin. This study proves that curcumin loaded microsponges may act as a promising drug delivery system for treatment of ulcerative colitis.

## 1. Introduction

Inflammatory bowel diseases (IBDs) are idiopathic inflammatory and relapsing disorders of the digestive tract. Ulcerative colitis (UC) and Crohn's disease are the two major chronic IBDs involving the large intestine or colon [1, 2]. Conventional drug therapy to treat IBD mainly includes the use of anti-inflammatory drugs which encounters obstacle such as absorption and degradation in the upper GIT and also causes serious side effects in upper GIT [3]. Thus, in the last decade colon-targeted drug delivery systems have gained significant interest due to their well-documented advantages such as specific drug targeting, reduction of doses and systemic side effects, and enhanced drug efficacy [4]. Microsponge delivery system (MDS) is the unique technique which provides targeted and controlled release of drug. Another added advantage of microsponges which contributes to their use is the ability of retaining drug on the surface of colon ensuring local and targeted action. Consequently, the lag time for drug absorption increases which makes MDS suitable for colon specific delivery [5]. MDS is highly porous, polymer based, cross linked, spongy spheres that have the ability to entrap and adsorb a wide range of active ingredients like anti-inflammatory, antifungal, antimicrobial, emollient, essential oils, and so forth. Microsponges have particle size ranging from 5 to 150 *μ*m and are highly efficacious, stable, nonirritant, nontoxic, nonallergic, nonmutagenic. It also reduces side effects and increases elegance and is compatible with the active ingredients [6]. Curcumin is a yellow-colored naturally occurring polyphenol obtained from the rhizomes of* Curcuma longa*. The safety and pharmacological efficacy of curcumin make it a suitable molecule for the prevention and treatment of various diseases. It is a highly pleiotropic molecule which can interact with different molecular targets involved in inflammation [7]. Curcumin also acts effectively against ulcerative colitis. It can modulate inflammatory response by downregulating the activity of cyclooxegenase-2 [COX-2], lipoxygenase, and inducible iNOS [nitric oxide synthase] enzyme [8, 9]. Curcumin is of natural origin and hence safe and nontoxic even at higher doses [10]. In last decade various colon targeting formulations of curcumin have been suggested by several researchers such as guar gum based tablets, microspheres, nanoparticles, and so forth. But these formulations have some drawbacks, which can be overcome by formulating microsponges as shown in Table 1. In this study we aimed at fabrication of curcumin loaded microsponges for colonic drug delivery. Curcumin loaded microsponges were prepared by quasi emulsion solvent diffusion method using Eudragit and water soluble porogen. Microsponges were optimized using 3^2^ full factorial design. The effect of independent variables on the particle size, entrapment efficiency, and % cumulative drug release was evaluated. Finally, the optimized formulation was subjected to* in vivo* study using acetic acid induced colitis model in rats.

## 2. Material and Methods

### 2.1. Materials

Curcumin was obtained as a gift sample from Konark Herbal and Health Care, Mumbai, India. Eudragit L100 was received from Degussa India Pvt. Ltd., and polyvinyl alcohol (PVA) was purchased from the Nice Laboratory Reagent. Other excipients used were of standard pharmaceutical grade and all chemicals and reagents used were of analytical grade.

### 2.2. Fabrication of Microsponges

Microsponges were prepared by quasi emulsion technique using porogen. A (1% aqueous solution of sodium chloride) was prepared which was used as porogen. A sufficient amount of Span 80 was added to the prepared porogen with agitation to obtain 1% (v/v) dispersion. A solution of curcumin and Eudragit L100 [11] was prepared in ethanol. Then porogen solution was uniformly emulsified in curcumin and Eudragit solution. 5% w/v aqueous PVA solution was prepared separately. Then the earlier prepared dispersion was emulsified in PVA solution. This emulsion was continuously stirred for 3 h to get microsponges. Then the microsponges were filtered and dried at 60°C and stored in desiccators [12]. Microsponges were prepared based on 3^2^ full factorial design and total nine formulations were prepared [F1–F9]. Each formulation varied by volume of ethanol and amount of Eudragit at three different levels (Table 2).

### 2.3. *In Vitro* Characterization of Microsponges

#### 2.3.1. Determination of Particle Size, Shape, and Surface Morphology

The curcumin loaded microsponges formulations were analyzed for determination of average diameter by Zeta-Sizer (Malvern Instruments, Mastersizer 2000, UK). The values (*d*_50_) were denoted for all formulations as average size range [13].

The microsponge formulations were visualized by scanning electron microscope (SEM) to assess the morphology of the microsponges and surface. Samples were coated with gold-palladium under an argon atmosphere at room temperature and the morphology of the microsponges was studied with SEM at 10 kV (QUANTA 250, FEI Makers, Singapore) [14].

#### 2.3.2. Determination of Encapsulation Efficiency, Percentage Yield, and Drug Loading

Curcumin microsponges [100 mg] were crushed and extracted using 10 mL methanol by vortexing and centrifuging at 2000 rpm for 10 min. Then insoluble residue was separated and the supernatant was analyzed spectrophotometrically at 430 nm after appropriate dilution. Then, the encapsulation efficiency, percentage yield, and drug loading were calculated by the following equation [15]:

(1) Encapsulation  efficiency  (EE)  =(Mass  of  drug  in  microspongesInitial  mass  of  drug)×100,Percentage  yield  (PY)  =(Mass  of  obtained  microspongesInitial  mass  of  drug+Initial  mass  of  polymer)   ×100,Drug  loading  (DL)  =(Mass  of  drug  in  microspongesMass  of  microsponges)×100.

### 2.4. *In Vitro *Drug Release

Dissolution study of curcumin microsponges was carried out in USP dissolution test apparatus II stirred at 100 rpm and temperature of 37 ± 0.5°C (Paddle type, Electro lab. EDT-08 11LX). Drug release was monitored for 12 h and samples were withdrawn periodically and sink conditions were maintained by replacing with equal amount of fresh dissolution medium. The dissolution was carried out at different pH condition using 0.1 N HCl (pH 1.2) for 2 h, phthalate buffer (pH 4.5) for next 2 h, and phosphate buffer (pH 6.8) with 4% w/v fresh rat caecal content (RCC) under anaerobic condition for subsequent hours to simulate the GIT condition. After 12 h study, the samples were analyzed by UV spectrophotometer at 430 nm [16, 17].

### 2.5. Optimization of Experimental Design

The effect of independent variables (volume of ethanol and Eudragit L100) on the dependent variables (% EE, % cumulative drug release, particle size) was modeled by Design Expert software version 8.0.7.1 [18]. Further theoretical formulation F10 was designed as an extra checkpoint to validate the predictability of optimization study (Table 2).

### 2.6. *In Vivo* Study

#### 2.6.1. Acetic Acid Induced Experimental Ulcerative Colitis in Colon

Wistar Albino rats (body weight = 160–200 g), *n* = 5, were selected and were caged individually with food and water* ad libitum*. All the studies were conducted with prior approval of Institutional Animal Ethical Committee (IAEC/SU-PHARM/13/006).

The rats were distributed randomly in three groups, that is, control, curcumin, and microsponges of curcumin, each comprising five animals. 1 mL (4%) (v/v) of acetic acid was given to induce ulcerative colitis in rats through intrarectal route which resembled with the inflammatory bowel disease in all groups [19, 20]. For three days, rats were housed without any treatment to maintain the development of full IBD model. Each group received the treatment orally in 1% carboxymethyl cellulose (w/v) solution. Group 1 received vehicle only, group 2 received pure curcumin (20 mg/kg), and group 3 received microsponges of curcumin (20 mg/kg) [21, 22].

#### 2.6.2. Pharmacological Assessment

After 24 h of last drug administration, the animals were sacrificed, colon part was cut, and ulcer projections were visualized and assessed on the basis of the inflammatory scales; that is, 0 = normal colored colon, 0.5 = red coloration, 1 = spot ulcer, 1.5 = hemorrhagic streaks, and 2 = hemorrhagic ulcer. Histopathological studies were also performed by preserving the colonic part in 10% formalin solution [23].

### 2.7. Stability Study

The stability of curcumin microsponges (F7) was carried out as per ICH guidelines in accelerated conditions. The microsponge formulation was kept at 40°C ± 2°C and 75% ± 5% RH for three months. After 3 months microsponges were analyzed for physical appearance,* in vitro* drug release, and FTIR spectroscopy.

## 3. Results and Discussion

As stated in Section 2, microsponges were prepared by the quasi emulsion technique and subjected to various evaluation parameters.

### 3.1. *In Vitro* Characterization of Microsponges

Prepared microsponges were characterized for physical appearance, microscopic evaluation, particle size, encapsulation efficiency, percentage yield, drug loading,* in vitro* drug release, and pharmacodynamic activity.

### 3.2. Particle Size, Shape, and Surface Morphology of Curcumin Microsponges

The particle size of developed microsponges was analyzed by Zeta Sizer, which showed the average size of microsponges was within the range of 41.63 *μ*m to 57.59 *μ*m. The particle size of microsponges was affected by volume of solvent and concentration of Eudragit, as increase in volume of ethanol produces less viscous solution, which resulted in smaller particle size, as exhibited by F7 (41.63 *μ*m), F8 (45.62 *μ*m), and F9 (47.23 *μ*m) (Table 3).

**Figure 1:**
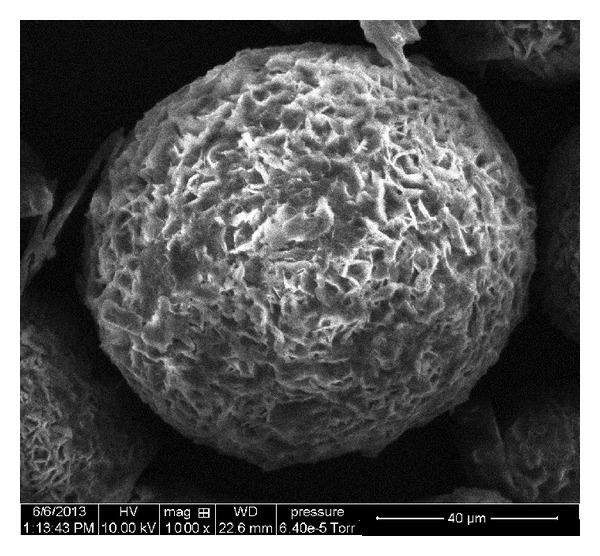
SEM photomicrograph of curcumin microsponges.

The microsponges' formulation F7 was visualized by scanning electron microscope to assess the morphology of microsponges. SEM image revealed spherical and porous surface as shown in Figure 1.

### 3.3. Determination of Encapsulation Efficiency, Percentage Yield, and Drug Loading

Table 3 shows the values of drug loading, percentage yield, and encapsulation efficiency of different batches of curcumin loaded microsponges. The PY values range between 71.49 and 88.89%. The different formulations showed EE values varied between 66.12% and 78.13%. The EE was found to increase with increase in volume of ethanol that may be due to the higher solubilization of drug in ethanol. Higher volume of solvent resulted in uniform mixing of drug and solvent which resulted in higher EE. Moreover, particle size of microsponges also affected the encapsulation efficiency. EE was found to increase with decrease in the particle size. This might be due to the fact that smaller particle size will offer more surface area for adsorption of drug. F7 formulation having smallest particle size (41.63 *μ*m) showed highest EE of 78.13%.

### 3.4. *In Vitro* Release Study of Curcumin Microsponges

**Figure 2:**
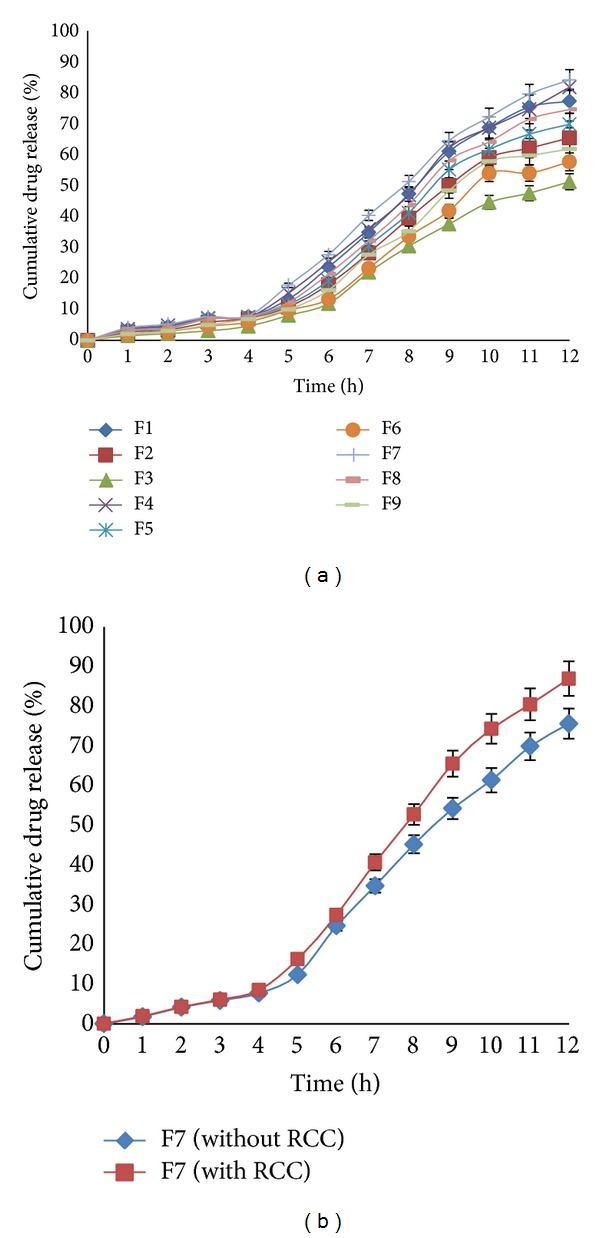
(a) Comparative dissolution profile of curcumin loaded microsponges (F1–F9). (b)* In vitro* release profile of F7 with and without RCC.

*In vitro* release study of curcumin microsponges was carried out at different pH (pH 1.2 for 2 h, pH 4.5 for next 2 h, and pH 6.8 with 4% w/v fresh rat caecal content under anaerobic condition for subsequent hours) to mimic the GIT environment. The studies were carried out in 900 mL of the dissolution medium at 37 ± 0.5°C. The* in vitro* release of drug from microsponges is shown in Figure 2(a). Results of* in vitro* drug release revealed that 4.53 to 7.91% of CUR was released from the microsponges in initial 4 h and 51.25 to 84.12% of drug was released after 12 h. The drug release suggested that Eudragit L100 prevented the premature release of curcumin in the upper GIT, since it is a pH sensitive polymer having threshold pH value above 6, which bypasses the GIT and showed drug release above pH 6. Release rate of curcumin from microsponges increased after 4 h, due to the exposure of formulations to pH 6 which is above the solubilizing pH of Eudragit L100 polymer [11, 24]. Eudragit L100 also increased the solubility of curcumin by 62.87%. It was due to decrease in the particle size of drug to molecular level. Formulations F7, F4, and F1 exhibited higher drug release as compared to other formulations due to lower level of Eudragit.* In vitro* release of drug from F7 in presence and absence of RCC is depicted in Figure 2(b). Results revealed that in presence of RCC the release of drug from the microsponges was slightly higher, which might be due to the erosion of polymeric matrix in presence of RCC.

### 3.5. Optimization of Experimental Design

**Figure 3:**
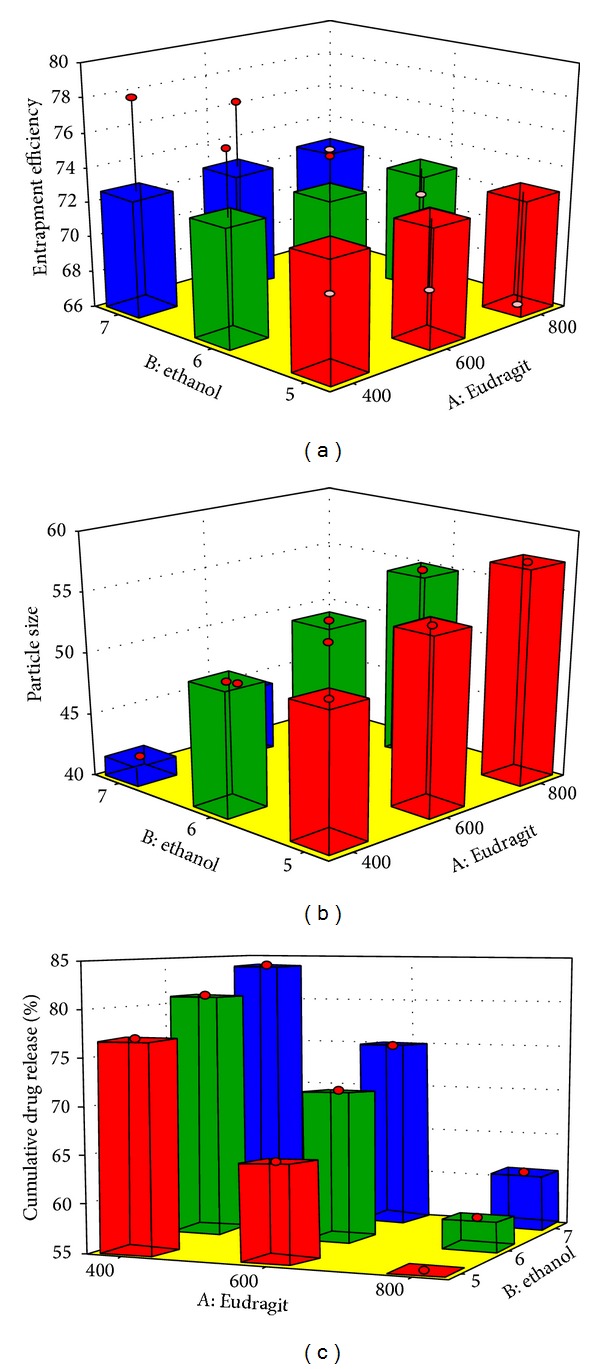
3D bar surface chart depicting the influence of independent variables over dependent variables.

The optimization studies were performed by using Design Expert software version 8.0.7.1 and second order polynomial equation was derived after transformation of variables (Figure 3). Further theoretical formulation F10 was designed as an extra checkpoint. The experimental value of % cumulative drug release (CDR) was compared with predicted value and there was no significant difference between the two values with similarity factor of 92.56 (*P* < 0.05), thus confirming the validity of optimization. The transformed equations are as follows. Equation (2) represented equation for drug release, (3) is for particle size, and (4) is for entrapment efficiency:

(2) $$\begin{array}{c} Y=62.9+1.45X_{1}+11.1X_{1}^{2}+8X_{2}-7.51X_{2}^{2}+1.85X_{1}X_{2}, \end{array}$$

(3) Y=52.8−4.51X1−2.48X12+2.86X2−0.52X22+0.30X1X2,

(4) Y=68.91+4.69X1−2.8X12−2.01X2−1.65X22−1.03X1X2.

### 3.6. Selection of Optimized Formulation

F7 formulation was identified as optimized formulation based on the smallest particle size (41.63 ± 0.25 *μ*m), the highest EE (78.13 ± 1.45%), the highest drug release (84.12%), and desirability factor of 0.83. Further the F7 formulation was used for pharmacodynamic evaluation. The drug release profile of F7 formulation was subjected to different kinetic models and based upon the best correlation coefficient (*r* ^2^ = 0.9927) the release was found to follow Higuchi model, which suggested diffusion as the main mechanism of drug release.

### 3.7. *In Vivo* Study

*In vivo* study using acetic acid induced colitis model in rat showed that ulcers in curcumin loaded microsponges treated group were recovered up to a good extent as compared to control group while in pure curcumin treated group the healing was mild. Results are shown in Table 4. Sections of colon were also assessed for histopathological studies.

### 3.8. Histopathology Studies

**Figure 4:**
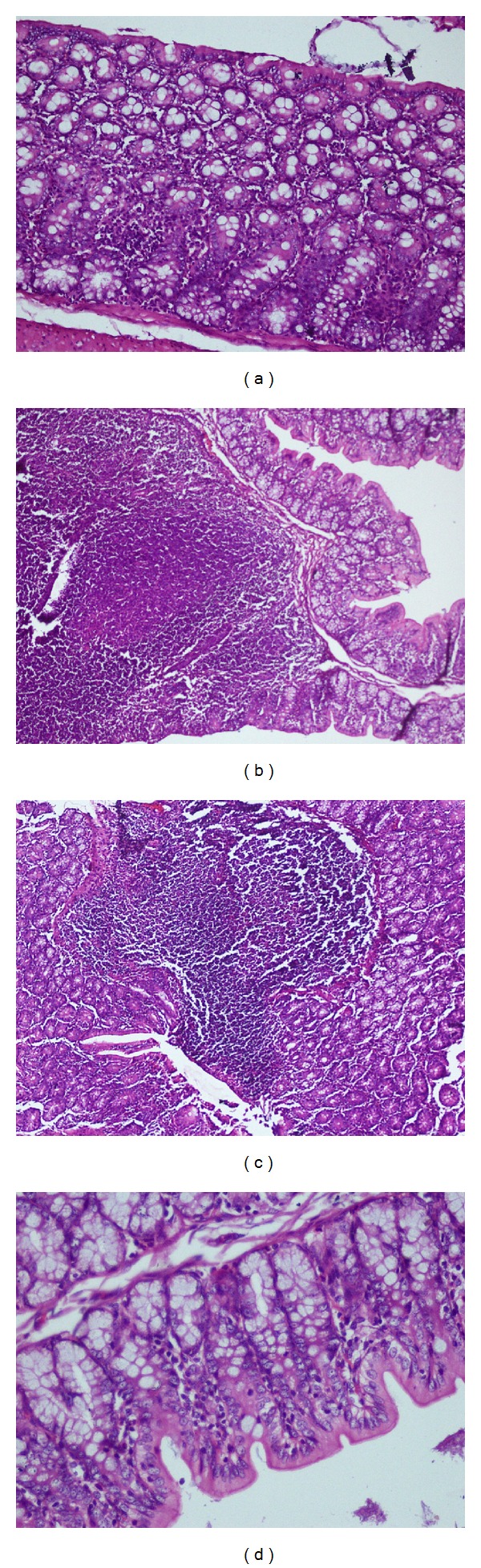
Histology of colonic section of (a) normal group, (b) acetic acid induced colitis group, (c) curcumin treated group, and (d) curcumin loaded microsponges treated group.

After examining the sections of the colon of the rat, it was revealed that acetic acid induced edema in submucosal layer and caused hemorrhage, necrosis, and intense inflammation on colon (Figure 4(b)). While in case of normal rat colon, there was no inflammation as shown in Figure 4(a). Curcumin treated group showed decrease in ulceration, necrosis, and hemorrhage up to a moderate extent as shown in Figure 4(c). However, the curcumin microsponges treated group showed significant decrease in these pathological parameters as compared to free curcumin (Figure 4(d)). These results suggested that the microsponges of curcumin may be used as a promising drug delivery system for treatment of ulcerative colitis.

### 3.9. Stability Studies

**Figure 5:**
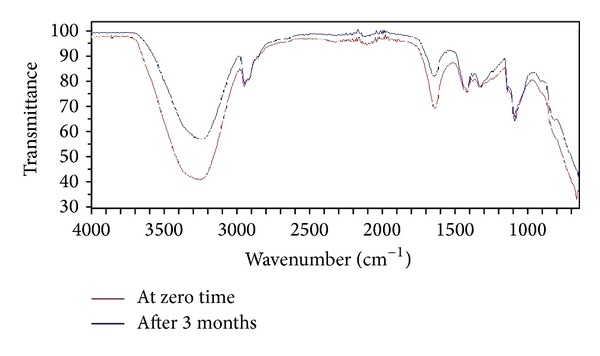
FTIR spectra depicting stability of developed formulation.

The F7 formulation was subjected to 3-month stability study at accelerated condition and was analyzed for physical appearance,* in vitro* drug release, and FTIR spectroscopy. After 3 months the formulation was found to show no change in physical appearance and* in vitro* drug release (similarity factor 91.45). The FTIR spectra revealed no sign of instability (Figure 5). Thus, all these parameters suggested that the formulation F7 may have good shelf life.

## 4. Conclusion

Curcumin loaded microsponges were successfully developed by quasi emulsion technique for colon targeting. The developed formulation was found to be spherical with tiny pores on the surface. Eudragit L100 was used as a pH sensitive polymer having threshold pH value above 6, which bypassed the upper GIT and showed targeted and controlled release at colonic pH as revealed by* in vitro* drug release study. The* in vivo* studies also revealed better therapeutic outcomes as compared to free curcumin. Thus the results suggested that curcumin loaded microsponges may be considered as a promising drug delivery system for treating ulcerative colitis.

## Figures and Tables

**Table 1:** Disadvantages of various curcumin colon targeted formulations.

Formulation	Disadvantage
Guar-gum based tablet	Single unit system has disadvantage of unintentional disintegration of tablet in GIT, which may lead to compromised systemic bioavailability.

Microspheres and nanoparticles	Microspheres and nanoparticles are comparatively nonporous in nature and have less capability to bind to the rough surface of intestinal mucosa as compared to microsponges particles.

**Table 2:** 3^2^ factorial design for preparing curcumin loaded microsponges.

Formulations	Drug (mg)	PVA (g)	Eudragit (mg)	Ethanol (mL)
F1	800	5	400	5
F2	800	5	600	5
F3	800	5	800	5
F4	800	5	400	6
F5	800	5	600	6
F6	800	5	800	6
F7	800	5	400	7
F8	800	5	600	7
F9	800	5	800	7
F10∗	800	5	700	6.5

*Extra design checkpoint.

**Table 3:** Compilation of various evaluation parameters for curcumin microsponge formulations.

Formulation code	Particle size* (*μ*m)	EE (%)*	PY (%)*	DL (%)*
F1	51.06 ± 0.15	70.36 ± 1.23	87.26 ± 0.42	37.71 ± 0.22
F2	54.44 ± 0.96	68.75 ± 0.98	88.89 ± 0.92	40.18 ± 0.56
F3	57.59 ± 0.55	66.12 ± 1.96	74.45 ± 0.74	45.36 ± 0.74
F4	50.14 ± 0.71	76.61 ± 0.84	86.13 ± 1.55	69.45 ± 0.98
F5	52.95 ± 0.22	74.85 ± 0.56	86.39 ± 1.78	40.33 ± 1.20
F6	55.29 ± 0.81	71.19 ± 1.10	80.44 ± 0.71	64.45 ± 0.77
F7	41.63 ± 0.25	78.13 ± 1.45	80.15 ± 0.84	66.94 ± 0.68
F8	45.62 ± 0.69	76.74 ± 0.73	84.72 ± 0.58	47.69 ± 1.73
F9	47.23 ± 0.74	72.58 ± 2.31	71.49 ± 1.19	39.89 ± 0.84

*n = 3, mean ± S.D.

**Table 4:** Macroscopic evaluation of colonic lesions of rat.

Colonic erosion score*					
Groups	**0**	**0.5**	**1**	**1.5**	**2**
Control	—	1	1	2	1
Curcumin	—	1	3	1	—
Curcumin microsponges	—	3	2	—	—

*n = 5 in each group; 0 = normal colored colon, 0.5 = red coloration, 1 = spot ulcer, 1.5 = hemorrhagic streaks, and 2 = hemorrhagic ulcer.
